# Edge Caching in Fog-Based Sensor Networks through Deep Learning-Associated Quantum Computing Framework

**DOI:** 10.1155/2022/6138434

**Published:** 2022-01-07

**Authors:** Tayyabah Hasan, Fahad Ahmad, Muhammad Rizwan, Nasser Alshammari, Saad Awadh Alanazi, Iftikhar Hussain, Shahid Naseem

**Affiliations:** ^1^Department of Computer Sciences, Kinnaird College for Women, Lahore 54700, Punjab, Pakistan; ^2^Department of Basic Sciences, Deanship of Common First Year, Jouf University, Sakaka 72341, Aljouf, Saudi Arabia; ^3^Department of Computer Science, College of Computer and Information Sciences, Jouf University, Sakaka 72341, Aljouf, Saudi Arabia; ^4^Division of Engineering Management and Decision Sciences, College of Science and Engineering, Hamad Bin Khalifa University, Doha 34110, Qatar; ^5^Department of Information Sciences, Division of Sciences and Technology, University of Education, Lahore 54770, Pakistan

## Abstract

Fog computing (FC) based sensor networks have emerged as a propitious archetype for next-generation wireless communication technology with caching, communication, and storage capacity services in the edge. Mobile edge computing (MEC) is a new era of digital communication and has a rising demand for intelligent devices and applications. It faces performance deterioration and quality of service (QoS) degradation problems, especially in the Internet of Things (IoT) based scenarios. Therefore, existing caching strategies need to be enhanced to augment the cache hit ratio and manage the limited storage to accelerate content deliveries. Alternatively, quantum computing (QC) appears to be a prospect of more or less every typical computing problem. The framework is basically a merger of a deep learning (DL) agent deployed at the network edge with a quantum memory module (QMM). Firstly, the DL agent prioritizes caching contents via self organizing maps (SOMs) algorithm, and secondly, the prioritized contents are stored in QMM using a Two-Level Spin Quantum Phenomenon (TLSQP). After selecting the most appropriate lattice map (32 × 32) in 750,000 iterations using SOMs, the data points below the dark blue region are mapped onto the data frame to get the videos. These videos are considered a high priority for trending according to the input parameters provided in the dataset. Similarly, the light-blue color region is also mapped to get medium-prioritized content. After the SOMs algorithm's training, the topographic error (TE) value together with quantization error (QE) value (i.e., 0.0000235) plotted the most appropriate map after 750,000 iterations. In addition, the power of QC is due to the inherent quantum parallelism (QP) associated with the superposition and entanglement principles. A quantum computer taking “*n*” qubits that can be stored and execute 2^*n*^ presumable combinations of qubits simultaneously reduces the utilization of resources compared to conventional computing. It can be analyzed that the cache hit ratio will be improved by ranking the content, removing redundant and least important content, storing the content having high and medium prioritization using QP efficiently, and delivering precise results. The experiments for content prioritization are conducted using Google Colab, and IBM's Quantum Experience is considered to simulate the quantum phenomena.

## 1. Introduction

Fog computing (FC), at the edge of a sensor network, as an extension to cloud computing, offers storage, processing, and communication control services [1, 2]. In the period of next-generation telecommunication and through the massive development of the Internet of Things (IoT) based smart devices, applications required ultralow latency because IoT networks induce strain not only on the backhaul but the fronthaul causing adverse situations for interruption sensitive applications [3, 4]. These problems can be resolved through FC, which provides distributed computing and communication facilities from centralized servers in the edge direction. A central base band unit (BBU) pool is not robust for every control, communication, or any other processing function; therefore, FC-based radio access networks (F-RANs) were introduced. In F-RANs, the local BBUs or even remote radio heads (RRHs) are dedicated to such tasks through edge caching (EC) [5–9]. Due to an intermediate fog layer between end-users and the cloud, mobile edge computing (MEC) is introduced.

Although the idea of F-RANs seems to be propitious to provide all the tasks confronted by the cloud radio access networks (CRANs) or heterogeneous cloud radio access networks (HCRANs). However, some setbacks might cause performance deterioration or the quality of service (QoS) degradation, bringing about fronthaul congestion. The main issue that requires to be solved is EC as well as restricted storing capability in RRHs [10]. Limited vital interests associated with the EC trending in F-RANs are reducing fronthaul burden, backhaul, or even backbone, optimizing endwise latency issues, and dynamic applications of content responsive approaches performance improvements. Fog access points (FAPs) usually have a relatively minimal caching capacity mostly because of the limited memory linked to the caching processes executed in centralized CRANs or HCRANs. Nevertheless, an increase in cache size in the base stations (BS) has a balance in the middle of improved throughput and network spectral efficiency [11]. Consequently, caching techniques in FAPs, together with caching strategies and allocation of cache resources policies, need to be managed logically and dynamically for augmented F-RAN performance.

The main contribution of this research is to predict the high priority content through the deep learning (DL) technique. It is the leading task that must be carried out when the contents are requested repeatedly and placed in the caches. The rest of the content should be discarded. When the high priority content is predicted through the DL agent, efficient content management and placement are achieved through the proposed framework and the quantum memory modules (QMM) to store the content. This paper describes an EC-based deep learning-associated quantum computing (DLAQC) framework. The framework is based on two parts: one for caching content prioritization and the other one for caching content stored within the edge. The DL-based quantum computing (QC) approach associated with quantum information processing is deployed to enhance the performance of F-RANs. The framework is basically a merger of a DL agent deployed at the network edge and a QMM. Firstly, the DL agent prioritizes caching contents via Self-Organizing Maps (SOMs) algorithm, and secondly, the prioritized contents are stored in QMM using a Two-Level Spin Quantum Phenomenon (TLSQP). SOMs algorithm is staunchly suitable to pick up contents in colored cluster form without reducing the dimensionality of the feature space.

The paper's organization is as follows.  Section 2 describes the related work for edge caching, SOMs applications, and Stern–Gerlach experiment (SGE). In Section 3, the framework and algorithm are described, followed by an overview of the model. The DL agent in edge and TLSQP is also discussed. The experiments and results are analyzed and discussed in Section 4. Finally, conclusion is presented in Section 5.

## 2. Literature Review

In this section, a literature study is carried out to throw light on attempts of different researchers to enhance EC to improve efficiency and quality of service (QoS) in F-RANs. Several pieces of research related to the DL-based algorithm and SGE highlight their applications in various fields, which has proved to be a great source of guidance for the proposed idea.

The authors in [12] described the key features of MEC, especially in the context of next-generation and IoT-based applications. The role of MEC and its challenges in the context of edge intelligence is also described. By keeping in view, the latency, context awareness, and energy-saving criteria, it is compared with the conventional MCC by considering the following key enabling features: virtual reality/augmented reality, software defined network, network function virtualization, smart devices, information-centric networking, network slicing, and computation offloading. Additionally, a use case is also provided to help understand the edge intelligence in the IoT-based scenarios.

The critical challenges in the F-RANs are (i) the content placement in caches and (ii) the joint user associations. These challenges are tinted due to the complexity of different approaches used to find optimal solutions. In [13], authors considered optimization problems as mixed-integer nonlinear programming (MINL). A hierarchical game theory approach is applied, and a series of deep reinforcement learning (DRL) based algorithms are designed for user association, content popularity prediction, and content placement to enhance the FAPs. In [14], a cooperative EC scheme using the DRL approach to place and deliver contents in vehicular edge computing networks is presented. The deep deterministic policy gradient algorithm provides a sensible solution for long-term MINL problems. A scheme for vehicle scheduling and bandwidth allocation is designed to make it less complex to manage adaptive resources and make decisions.

A user preference-based learning EC policy is described in [15] to predict the online content popularity and an offline learning algorithm. A sigmoid function is exploited to construct a logistic regression model to estimate user preferences regarding online content popularity prediction. It is considered complicated to make a preference model for each user due to the high-dimensional feature space. Therefore, a follow-the-regularized-leader proximal inspired algorithm is also proposed for offline user preference learning.

The federated learning (FL) framework and deep reinforcement learning (DRL) techniques were integrated into MEC in [16] to optimize EC, computing, and communication. The key challenge is primarily faced by authors to place the DRL agents due to (i) the massive and redundant data transmissions in the cloud and (ii) the privacy risks as well as the lesser computing capabilities in UEs. The proposed technique has outperformed the conventional caching policies for EC like Least Recently Used, Least Frequently Used, and First In First Out. However, for computation offloading, the DRL technique by FL offered close results to the centralized DRL technique; on the other hand, again, it outperformed the following baseline policies: mobile execution, edge node execution, and greedy execution.

The authors in [17] have presented an on-demand and collaborative deep neural networks (DNN) coinference framework. The presented framework worked in two ways. Firstly, the DNN computation is partitioned between devices and the edge to make use of hybrid computation resources, and it can exit early in the DNN right-sizing at some suitable intermediate DNN layer. Therefore, it can avoid further computation latency and have implemented their prototype on Raspberry Pi. Secondly, the visualization of higher-dimensional data is taken into account to effectively analyze and conclude the data and results. The following two strategies have been used to visualize the multidimensional and minimal data by a scatter plot established on dimensionality reduction: (i) the direct visualization and (ii) the projection methods.

Failure modes and effects analysis (FMEA) is a methodology for risk analysis and problem prevention by identifying and defining failures of the system, process, or service. FMEA has some shortcomings related to the worksheet and usage complexity, which have been dealt with by the SOMs algorithm in [18]. SOMs algorithm is exploited to achieve perceptibility for corrective actions. A risk priority interval is used to evaluate these corrective actions in groups to make it easier for the users.

SOMs for multiple travelling salesman problem (MTSP) with minmax objective is exploited for the robotic multigoal path planning problem [19]. The main issue in deploying this framework was to detect the collision-free paths to evaluate the distances in the winner selection phase. The collision-free path was needed to adapt the neurons to the presented input signals. To address this issue, simple approximations of the shortest path are considered and verified through cooperative inspection. The presented SOMs approach is used to solve this inspection task by MTSP-minmax and compared with the MTSP-GENIUS algorithm.

In [20], the SOMs have been used to classify astronomical objects like stars' stellar spectra. The algorithm is used to make different spectral classes of the Jacoby, Hunter, and Christian library. The 158 spectra were chosen to classify by 2799 data points each. 7 clusters were formed from O to M, and 12 out of 158 spectra were misclassified, giving a 92.4% success rate. Otto Stern and Walther Gerlach, in 1922, performed an experiment that separated an electron beam while passing through a nonuniform magnetic field. When a beam passed through a magnetic field, two distinguished beams were observed on the screen. The experiments conducted by Otto Stern and Walther Gerlach gained popularity and were used in multidisciplinary studies by researchers.

In [21], the SGE is exploited in physical chemistry to investigate the magnetic response of the Fe@Sn12cluster. A comparison is carried out between Mn@Sn12 and Fe@Sn12 clusters by passing their beams through magnetic fields separately. The molecular beam of Fe@Sn12 cluster exclusively deviates more towards increasing the magnetic field. The beam deviates even at the shallow temperature due to the distortions of tin-cage induced by Jahn-Teller. Hence, in the magnetic dipole moment, the role of electronic orbital angular momentum is significant. The magnitude of the magnetic dipole moment is calculated from the transfer of the beam.

In [22], the SGE is oppressed to explore the spin ½ neutral particles' motion and how their motion is dependent on the initial phase difference between two internal spin states. If the particles are moving horizontally, the initial phase difference between spin states results in particle splitting in the longitudinal direction and in the lateral direction due to the quantum interference. This interference provides an alternate way of measuring the initial phase difference between spin states and helps determine the amplitude and phase of atoms in the same SGE. To study this phenomenon, the ultracold temperature is maintained to make the ideal condition for the atom to behave like a quantum wave packet instead of a particle. In general, an atom is not in a pure state, rather a mixed state and cannot be characterized as a single wave function.

The content priority is deduced by the adaptive neurofuzzy inference system (ANFIS) in [23] in which the following five input variables were carefully selected: video_elapsed_time, video_size, views, likes, and downloads. Each input variable has three membership functions having priorities high, medium, and low, and fifteen similar functions are made in the Sugeno inferencing mode. A rule base function was also created. After content prioritization through ANFIS, a theoretical explanation of the SGE is specified as a TLSQP for storing the prioritized content in quantum repositories.

Considering media requirements explicitly, EC seems to alleviate certain challenges. Occasionally, multimedia contents get heavier than even the encyclopedias, resulting in higher hardware and network capacities. EC can support such kinds of throughput requirements proportionally. Moreover, the scalability of streaming servers, which require special provisioning of these servers, can also be handled during live events. However, reducing the distance between end devices and BS will not be sufficient for increased network throughput; high-speed backhaul connectivity is also required between all the BS and the BBU Pool where centralized servers reside. The network traffic load can be reduced by minimizing redundant traffic. The traffic load mainly comprises content deliveries for the most requested/popular content at different times. If at all this redundant traffic is managed so that popular content is predicted and placed within the edge, the idea's effectiveness to increase network efficiency can be justified. Researchers and network specialists also have incorporated different AI techniques, including machine learning, to minimize the redundancy of network traffic and optimize the overall network efficiency, from predicting popular content to optimizing specific parameters and much more [24–27]. In this digital era, IoT-based devices have generated an enormous amount of data daily, which is one reason for the possible growth of DL algorithms [28]. The DL algorithms require a massive amount of data to learn from.

## 3. Deep Learning-Associated Quantum Computing Framework

Edge computing acts as an intermediary between cloud and user equipment through the network edge. Researchers and engineers are continuously trying to accelerate content deliveries further and make mobile services better. The intelligence of edge systems is enhanced by introducing a DL agent in network edge. The DL agent is used to prioritize (i) the caching content according to its popularity determined by the considered parameters and (ii) the content to be managed logically within the planned framework. Prioritizing the content intelligently for caching only is not adequate to optimize the overall performance of the system. The prioritized contents need to be stored efficiently in caches and accessed multiple times with instantaneous delivery response. Within edge caches, the TLSQP will take over to avoid limited storage issues.

### 3.1. Overview

**Figure 1:**
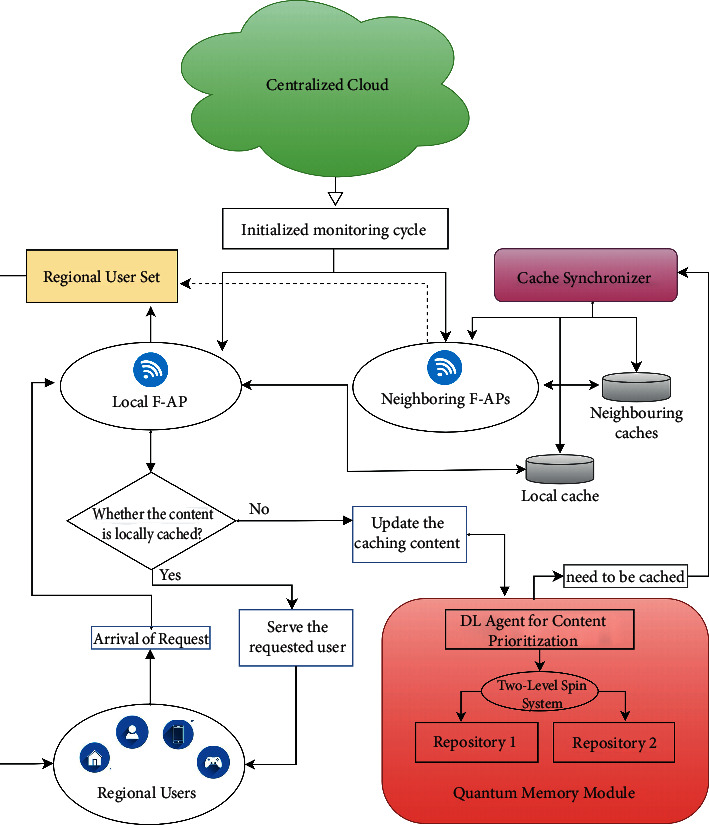
The proposed deep learning-associated quantum computing (DLAQC) framework.

To understand the workflow, a model is presented wherein the fog environment is described together with the proposed DLAQC framework and shown in Figure 1.

A particular region is considered from where user requests are generated. The cloud servers initialize the fog environment's monitoring cycle from the BBU pool. In the fog layer, at every moment, numerous user requests are generated and served through the F-APs. In order to accelerate content deliveries or response time, a DLAQC framework is presented, and a brief overview is as follows:

1. Synchronizer: cache synchronization and inter-F-APs information sharing is carried out every moment to update the regional user set for particular F-APs in the region
2. Regional user set: it refers to the group of users from a particular region allotted to a locally installed F-AP for a particular period.
3. Local and neighboring F-APs: the FAPs are capable of caching and computation and serve user requests by searching through the caches within the edge. A particular user request is immediately served if the content is available locally. The content must be looked up from the neighboring F-APs caches when a local cache is missed. In case if the contents are not available in the neighboring F-APs, the offered framework will update the respective contents in caches' QMM.
4. DL agent for content prioritization: in case of a cache miss, the content is intelligently updated through the DL agent deployed at the edge. It comprises SOMs and helps to predict the contents' priorities. The contents having maximum demand are considered highly prioritized. However, this module is used to logically manage the contents (specifically that need to be updated).
5. Quantum memory module: it is one of the most critical modules in the given setup. Once the contents are prioritized intelligently through the DL agent, the contents need to be stored optimally in caches to enable caching content management. The synchronizer module is used to do so. The requested content may also be served through (i) F-APs located in the same region or (ii) user dynamics or load balancing. The QMM is incorporated especially to place contents physically in caches as quantum particles when it is prioritized. As a case, in this model, Repository 1 is assigned for storing highly prioritized contents, and Repository 2 is assigned for the medium prioritized contents, respectively. QMM is used to store and serve (the requested) contents separately. Due to the lower demand, every time, the low-priority contents are discarded from the caches.

The proposed framework comprises two modules: DL agent and QMM. The DL agent is used to prioritize and logically manage the caching contents by making use of SOMs. The QMM is used to store the contents in a quantum regime by exploiting a TLSQP. The proposed framework's problem (working) and solution (implementation) domains are described as follows.

### 3.2. Framework (Problem)

#### 3.2.1. Deep Learning Agent in Edge

**Figure 2:**
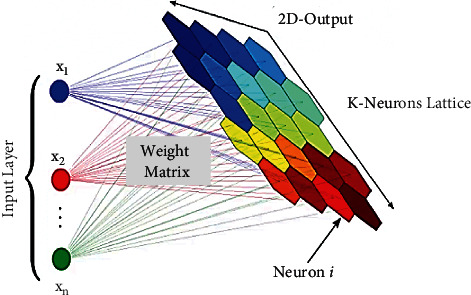
Structure of Self-Organizing Maps.

The DL agent is deployed at the network edge and prioritizes the caching contents through SOMs [29]. It gives results similar to the clustering approaches, and the prioritized contents can be visualized through light and dark color concentrations. The graphical output given by SOMs is a kind of feature map for input space. It makes SOM suitable for prioritizing the content using specific parameters. In this study, the media contents are explicitly targeted. Dataset for Trending YouTube Videos Statistics has been downloaded from Kaggle. The dataset includes the statistics for trending videos in the region of the United States. To achieve the requirements, four input variables for each video are carefully selected from the dataset and which are as follows: views, likes, dislikes, and comment_count. The identified relationship between input parameters is helpful for visualizing the trending contents' priorities using SOMs. The structure and function of SOMs are explained by the mathematical model as follows. SOMs work by fitting the map (grid of nodes) up to the given number of iterations of the simulated dataset. During diverse iterations, the adjustments are required while nodes' weights are adjusted to bring the map nodes closer to the data points. It is called the convergence of SOMs, and the structure for SOMs is given in Figure 2.

The main package is included to construct, evaluate, and visualize the map is Minisom. An input layer (4-dimensional) and feature space **M** of the map are defined by the rule of thumb. The rule of thumb states that there should be 5 · **sqrt**(**N**) neurons in the lattice to get desired results, where **N** is the total number of samples in the dataset (the training dataset). The training dataset has 40960 samples; thus, the lattice should contain $5\left(\sqrt{40949}\right)=1011\text{.}8$ neurons. Therefore, the dimensions of the lattice are selected as 32 × 32. Each node in the lattice has a weight vector **W**_**ij** _ and has the same dimensions as input vectors V. The preliminary step of training is to set weights of every node and is initialized as **W**_**ia** _: **W**_**ib** _: **W**_**ic** _: **W**_**i**  **d** _ where **i** represents node number and **a**, **b**, **c**, and **d** represent input vectors. When the weights are initialized, the best matching unit BMU is calculated by iterating through every node and by calculating the Euclidean distance between input vector **V** and each node's weight **W**. Finally, the smallest **W** is selected. The process is given as follows:

(1) $$\begin{array}{c} D=\;\sqrt{\overset{n}{\underset{i=0}{\sum }}{\left(V_{i}-W_{i}\right)}^{2}}. \end{array}$$

When the BMU is finalized, the neighborhood nodes whose weights need to be updated are determined. To achieve this, the Gaussian neighborhood function is used. In this function, the “bell-shaped curve” like weighting is considered to update the nodes depending upon their relative distances from the BMU. Initially, the sigma *σ*_0_ is used to denote the spread of the neighborhood function, and all nodes (come in this spread) are updated. The respective spread shrinks iteratively by using the function (decay function) described as follows.

(2) $$\begin{array}{c} \sigma t=\frac{\sigma_{0}}{\left(1+t\right)/\left(T/2\right)\;}, \end{array}$$

where **T** is the iterations set having {**t**_0_, **t**_1_, **t**_2_, **t**_3_,…, **t**_**n**_} and *σ*_**t**_ is the spread size at iteration **t**. Every node in the neighborhood of BMU is updated by

(3) $$\begin{array}{c} W_{\left(t+1\right)}=\;W_{t}+\;\Theta_{t}\left[L_{t}\left(V_{t}-W_{t}\right)\right]. \end{array}$$

A decaying function of learning is given as follows, where **L**_**t** _ is the learning rate.

(4) $$\begin{array}{c} L_{t\;}=\;\frac{L_{0}}{\left(1+t\right)/\left(T/2\right)\;}. \end{array}$$

Moreover, Θ_**t**_ is the distance effect from the BMU on the specific node and is given as follows:

(5) $$\begin{array}{c} \Theta_{t}=e^{-\left[D^{2}/2\sigma^{2}\right]}. \end{array}$$

Hence, blocks with similar color zones are visualized. Any new input will stimulate nodes in the zone with similar weight vectors. The process described above results in projection of all the data points onto the map that allows topology of high-dimensional input data to be preserved into two-dimensional output space. However, the visual inspection is not enough to determine (i) how well the map converges to the given data points or (ii) how well the map represents the underlying data. Some quality measures are developed to oblige the purpose of evaluating when the map is trained. Therefore, the Quantized Error (QE) is used for vector quantization to evaluate the quality of the map. It is achieved by summing up the distances between the nodes and the data points as per the average distance given as follows.

(6) $$\begin{array}{c} QE\left(M\right)=\frac{1}{N}\sum_{i=n}^{n}\left\|\varphi \left(x_{i}\right)-x_{i}\right\|, \end{array}$$

where the feature space of the map is denoted as **M**. **N** is used to represent the total number of data points and *φ*(**x**_**i**_) is used for mapping of data point x_i from input space to the map. Hence, it is considered as; the smaller the value of QE, the better it fits the data points. However, this quality measure can be used to compare maps by considering the same dataset and choosing the best one, not as the only quality assessment.

One of the primary aims of SOMs to determine quality is the topological preservation of high-dimensional input space in the two-dimensional output space. The topographic error (TE) is used to evaluate how well the individual data point is modeled to the map node by calculating the positions of 1^st^ BMU and 2^nd^ BMU. If these are located next to each other, the topology is preserved, and the TE is said to be zero for individual input. Similarly, summing up the errors for every input and calculating the data points as average are considered TE for the map as follows:

(7) $$\begin{array}{c} TE\;\left(M\right)=\frac{1}{N}\;\overset{n}{\underset{i=n}{\sum }}t\left(x_{i}\right), \end{array}$$

where **t**(**x**_**i**_) is a piecewise function; it is 0 if 1^st^ BMU and 2^nd^ BMU are neighbors or 1 otherwise. TE is evaluated to quantify the topology preservation by evaluating local discontinuities in the output map. Mostly, a tradeoff is realized between quality measures when increasing the projection quality and seems to decrease when some information is lost during this process.

#### 3.2.2. Quantum Phenomenon

*(1) Quantum Computing–Overview*. The QC is based upon physics' natural laws and claims to solve many (sub) atomic level problems that are inflexible for old style computers. Quantum parallelism is a distinctive feature established on superposition and entanglement and offers exponential acceleration of computation over conventional computers, especially for cryptosystems, making them acutely fast [30]. A quantum computer taking “*n*” qubits that can be stored and execute 2^*n*^ imaginable combinations of qubits simultaneously by joining them in an uncommon fashion recognized as superposition and defined as follows:

(8) $$\begin{array}{c} \;|\Psi \rangle =\alpha_{0}|0\ldots 00\rangle +\alpha_{1}|0\ldots 01\rangle +\cdots +\alpha_{2^{n}-1}|1\ldots 11\rangle , \end{array}$$

where *α*_*i*_*ɛ*  complex numbers known as probability amplitudes of qubits and ∑|*α*_*i*_|2=1.

In quantum information processing, electrons or photons in a coherent state is encoded with some required information (known as qubits) and pass to another qubit via a quantum bus. The passed information is accessible to many qubits in a system, accelerating the speed of computation, unlike classical computation [31, 32]. Trapped ion architecture, QC using superconducting qubits, and QC with nitrogen-vacancy center in diamonds are few of the hardware architectures considered for thorough research in well-equipped labs [33].

**Figure 3:**
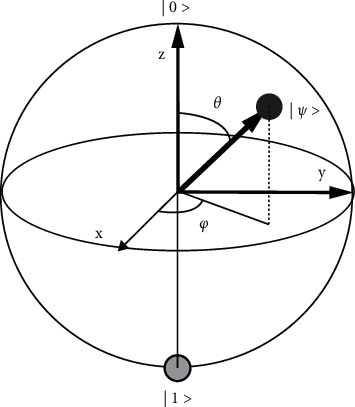
Representation of a qubit in a 2-dimensional Bloch sphere.

Like classical computation, quantum computation is carried out with the help of quantum gates. The information that has been obtained from quantum gates can be reversed. The representation of a single qubit quantum system is in the form of a Bloch (shown by Figure 3). Each quantum gate is represented by a matrix containing complex coefficients and can be applied on the qubit (state vector in Bloch sphere) to change state as a vector. A qubit is a state vector in two-dimensional Hilbert space. This vector can have any direction from the sphere's center to its periphery, i.e., it can connect to any point on the sphere's surface. The poles indicate the ground and excited states, and anywhere between these points is the superposed state of the qubit. Different qubit transitions indicate rotations about the axes changing the state of that qubit [34, 35]. Moreover, these rotations occur as a result of the quantum gate(s), which act on that qubit (see Figure 3).

*(2) Two-Level Spin System*. As described earlier, logical content prioritization is achieved through the DL agent to know about the requested content's priorities. Once the priority is known, the particle is encoded according to the relevant content priority. By taking into account the high and medium priorities, the contents can be stored physically in QMM by dividing it into two groups for which SGE is exploited. The information is encoded through the spin ±½ particles; +1/2 upward spin and −1/2 downward spin. The highly prioritized data are coded by spin-up particles, whereas through the spin-down particles, the likewise and medium prioritized data are encoded.

**Figure 4:**
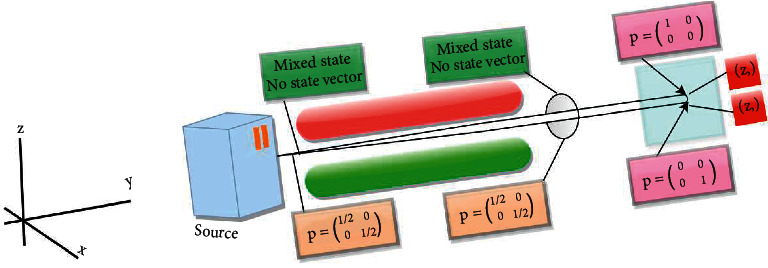
Electron beam splitting into two while passing through a magnetic field [23].

Due to the spinning environment, the electron has a magnetic field. The magnetic field can be canceled by another electron having an opposite spin in an atom. In an atom, the electrons are either paired or unpaired. To decide the spinning effect, the unpaired electrons leave the orbitals. Electrons are accrued like a beam, divided into two illustrious beams of equivalent power even though passing through a nonuniform magnetic field. Therefore, a massive tendency is shown in Figure 4.

A quantum organization is indicated by its state vector. But at times, the system is said to be in mixed state having statistical ensemble of various state vectors. Such a system has equal probabilities or chances to designate in either pure state. The pure state is basically a quantum state useful in the quantum system and determines the statistical behavior of the measurement. At the beginning of the TLSQP, all electrons are located in a mixed state since the states are indefinite. Due to the half-half chances of existence in any of the pure states (ǀ0〉 and ǀ1〉), the particles have a mixed state. Density matrices are used to represent the statistical state of the quantum system or a particle. The chances for result can be calculated from the density matrix for the system. The density matrices for states (i.e., mixed and pure) represented that the particles are initially in the mixed state when accrued like a single beam.

(9) $$\begin{array}{c} \rho =\left[\begin{array}{cc} \frac{1}{2} & 0\\ 0 & \frac{1}{2} \end{array}\right]. \end{array}$$

A nonuniform magnetic ground is formed by employing two magnets in a perpendicular way along the *z*-axis. When a beam passes through the magnetic field, electrons are bent alongside the axis comparative to the *z*-component. After passing through a magnetic field, some of the particles are in the Eigen state ǀZ+〉 of the Sz operator. The matrix for this state vector is given as follows:

(10) $$\begin{array}{c} \rho_{Z+}=\left|Z+\rangle |\langle Z+\right|=\left[\begin{array}{c} 1\\ 0 \end{array}\right]\left[\begin{array}{cc} 0 & 1 \end{array}\right]=\left[\begin{array}{cc} 1 & 0\\ 0 & 0 \end{array}\right]. \end{array}$$

The trace of this density matrix's square is 1, which clearly shows that it is a pure state. The state of the rest of the particles after passing through the magnetic field becomes 1 (ǀZ+〉). The matrix for this state is given as follows.

(11) $$\begin{array}{c} \rho_{Z-}=\left|Z-\rangle |\langle Z-\right|=\left[\begin{array}{c} 0\\ 1 \end{array}\right]\left[\begin{array}{cc} 0 & 1 \end{array}\right]=\left[\begin{array}{cc} 0 & 0\\ 0 & 1 \end{array}\right]. \end{array}$$

Again, this density matrix's trace is 1 and shows that it is a pure state. In contrast, if considered these two separated beams together, we again get the mixed state consisting of an equal mixture of particles in Eigen states ǀZ+〉 and ǀZ−〉, as follows:

(12) $$\begin{array}{c} \rho =\frac{1}{2}|Z+\rangle \langle Z+|+\frac{1}{2}|Z-\rangle \langle Z-|=\left[\begin{array}{cc} \frac{1}{2} & 0\\ 0 & \frac{1}{2} \end{array}\right]. \end{array}$$

The trace of the square of this density matrix is 1/2 which is less than 1, showing a mixed state. Before passing via a magnetic field, the electrons' spin can point in any direction of the space, being equally probable, so there is no state vector but for pure states.

The mined beams can be stored in QMM distinctly. The ǀZ+〉 state represents Repository 1 electrons devising pure state ǀ0〉 wherever (high) prioritized contents are stored. The ǀZ−〉 state signifies Repository 2 electrons taking pure state ǀ1〉, and the intermediate prioritized contents are stored. Therefore, electrons containing certain contents' information are categorized based on the established priorities by using TLSQP. The stated repositories help in storing the modified contents in every interval of time.

The ion-trap architecture of QMM is useful and effective for this specific scenario. The quantum data can be stored by qubits using atomic ions. The qubits (atomic ions) are trapped and designed by groupings of static and oscillating electric fields [33, 36, 37]. In what way, these quantum data are stored in these repositories which are beyond the scope of this research. The quantum information can be managed (processed or transferred) through the ions' cooperative quantized motion and is also recognized as quantum parallelism. The respective parallelism leads to an increase in the processing time as compared to the classical architectures. It has long been known that classical physics principles do not allow for causally efficacious understanding; yet, the intrinsic indeterminism and characteristic duality of quantum physics is that it contains give fertile ground for comprehension through physical modeling.

Measuring probability for spin-up and spin-down particles is an important aspect that will help determine the category of data encoded in a particular spin-type particle. So, to interpret the idea, IBM's QC simulator is exploited to yield some meaningful results. The resulting probability *p* for a particle to emerge as a spin-up particle can be found out by **cos**^2^(*θ*/2), and for a particle to emerge as spin-down can be found out by **sin**^2^(*θ*/2), where *θ* is the angle of rotation along *Z*-axis. Different angles of rotation will yield different likelihoods for spin-up and spin-down elements. The formula to find the probabilities of spin-up and spin-down elements is explained as the trace of density matrix and projection operator on that pure state-directed to some angle *θ* as follows:

(13) $$\begin{array}{c} p=\;Tr\;\left[\rho P_{\hat{n}}\right], \end{array}$$

where *ρ* is the density matrix of pure states already described above and $P_{\hat{n}}$ is the projection operator on a pure state which is directed to some $\hat{n}$ so that ${\hat{n}}_{\theta }=\left(cos\theta ,0,sin\theta \right)$. Hence,

(14) $$\begin{array}{c} P_{\hat{n}}=\frac{1}{2}\left[\begin{array}{cc} 1 & 0\\ 0 & 1 \end{array}\right]+\frac{1}{2}\left[\begin{array}{cc} 0 & sin\;\theta \\ sin\;\theta & 0 \end{array}\right]+\frac{1}{2}\left[\begin{array}{cc} cos\;\theta & 0\\ 0 & -cos\;\theta \end{array}\right]=\;\left[\begin{array}{cc} cos^{2}\frac{\theta }{2} & \frac{1}{2}sin\;\theta \\ \frac{1}{2}sin\;\theta & sin^{2}\frac{\theta }{2} \end{array}\right]. \end{array}$$

So, the trace of the product of *ρ* and $P_{\hat{n}}$ comes out to be **cos**^2^(*θ*/2) for │*Z*+〉 particles and **sin**^2^(*θ*/2) for │Z−〉 particles, respectively.

Quantum computers have stimulated the rotation produced by the magnetic field in the SGE by applying quantum gates. For instance, a **T** gate is used to produce rotation at *θ*=*π*/4. This gate rotates the state of the qubit in the superposed form by angle *π*/4 along the *Z*-axis. So, it is necessary to apply the Hadamard gate (**H**) before applying the **T** gate, as the **H** gate helps create superposition. Matrix representation of a Hadamard gate is shown as follows:

(15) $$\begin{array}{c} \frac{1}{\sqrt{2}}\left[\begin{array}{cc} 1 & 1\\ 1 & -1 \end{array}\right]. \end{array}$$

**Figure 5:**
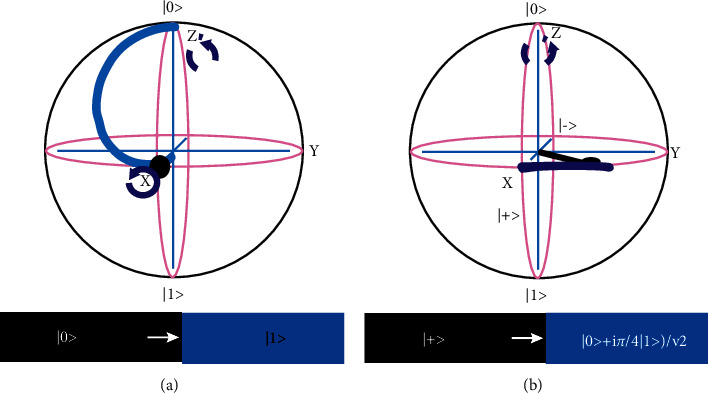
The gates are represented through a Bloch sphere. (a) Hadamard (H) gate representation in a Bloch sphere, (b) The rotation is produced by T gate and shown in a Bloch sphere.

It converts the │0〉 basis state of the qubit to $\left(\left(|0\rangle +|1\rangle \;\right)/\sqrt{2}\right)$ form (also known as │+〉), and │1〉 basis state to $\left(\left(|0\rangle -|1\rangle \;\right)/\sqrt{2}\right)$ form (also recognized as │−〉). Therefore, a superposition is created as there is an equal probability to be either 0 or 1. It produces two rotations simultaneously: *π* at the *z*-axis and *π*/2 at the *y*-axis and is shown by Figure 5(a).

After creating superposition, the **T** gate is applied to rotate the superposed qubit at *π*/4 along the *z*-axis. This is a single qubit gate (from the family of phase shift gates), which does not change the probability of the │*Z*+〉 and │Z−〉 somewhat changing the phase of the qubit's state. This gate acts on the │1〉 base state, whereas exiting the │0〉 base state remains unaffected. So, │+〉 will be converted to $\left(\left(|0\rangle +\iota \pi /4|1\rangle \;\right)/\sqrt{2}\right)$ and │−〉 will be converted to $\left(\left(|0\rangle -\iota \pi /4|1\rangle \;\right)/\sqrt{2}\right)$ because these are mixed states. Matrix representation of **T** gate is described as follows:

(16) $$\begin{array}{c} \left[\begin{array}{cc} 1 & 0\\ 0 & e^{\iota \pi /4} \end{array}\right]. \end{array}$$

The rotation produced through the **T** gate and is shown in Figure 5(b).

As soon as the **T** gate is applied, another **H** gate is again useful to maintain qubit's superposition. Alternatively, it would have lost its quantum state and collapsed into a classical one that is of course 0 or 1 depending upon the qubit chosen. The equation which satisfies this circuit is given as follows:

(17) $$\begin{array}{c} \frac{1}{2}\left[\begin{array}{cc} 1 & 1\\ 1 & -1 \end{array}\right]\left[\begin{array}{cc} 1 & 0\\ 0 & e^{\iota \pi /4} \end{array}\right]\left[\begin{array}{cc} 1 & 1\\ 1 & -1 \end{array}\right]\left[\begin{array}{c} 1\\ 0 \end{array}\right]=\left[\begin{array}{c} \frac{1}{2}+\frac{1}{2}e^{\iota \pi /4}\\ \frac{1}{2}-\frac{1}{2}e^{\iota \pi /4} \end{array}\right]. \end{array}$$

When more than one gate is applied on a qubit in a serially wired circuit, dot product (usual matrix multiplication) is carried out for all the gates, resulting in a combined gate acting on that qubit. As mentioned in equation (17), **H**, **T**, and **H** gates have been combined by dot product and applied on │0〉. The resultant matrix shows the probability amplitudes of spin-up and spin-down, as complex numbers, just before the measurement. It can also be written as follows:

(18) $$\begin{array}{c} \left(\frac{1}{2}+\frac{1}{2}e^{\iota \pi /4}\right)|0\rangle +\left(\frac{1}{2}-\frac{1}{2}e^{\iota \pi /4}\right)|1\rangle . \end{array}$$

The probability amplitude *α*^2^ of │0〉 is |(1/2)+(1/2)**e**^*ιπ*/4^|^2^ |^2^, equal to 0.8535534, approximately 85%, and that of │1〉 is the leftover probability which is of course 0.1464466 (approximately 15%). Hence, │0〉 basis state has a greater probability, so classically, a 0 is obtained by measuring the state if the **T** gate is applied. Similarly, some other appropriate sequence of **H** gates and phase shift gates can also be applied in order to produce a distinct rotation and obtain different probabilities of spin-up and spin-down particles. It depends upon which type of particle is needed to encode the data to be stored in the relevant repository.

A quantum computer can help to determine these complex probability amplitudes in terms of real numbers. It can then be classically interpreted and ultimately helping in encoding data.

### 3.3. Flowchart and Algorithm (Solution)

**Figure 6:**
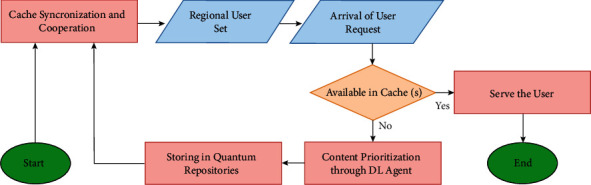
The flowchart of the proposed framework.

The flowchart of the proposed framework is represented in Figure 6. An algorithm is described (and also shown by Algorithm 1) as follows.

The algorithm comprises three functions: (1) cache_synchronization (2) cache_update , and (3) Serve_UE_Phase . The cache_synchronization function is used for cache synchronization and cooperation. It has parameters *t*, and *S*: *t* is the time interval after which cache information is shared, while *S* is a set of regional users for a particular *t*. It returns the regional user set for a particular time interval by considering the time interval, set of user requests, the workload on the edge node, and distance *d* of UE from the edge node. The information of *R*, *d*, and *w*_*E*_ at a specific time, quantum *t* is shared in Step 1. *R* is used for the set of user requests {*r*_1_, *r*_2_,…,  *r*_*k*_,…,  *r*_*n*_}, *d* is the distance of UE from edge node receiving a request, and *w*_*E*_ is the workload on a particular edge node. Step 2 will return a list of *S* for the particular *t*. In Step 3, assigned edge node **E**_**A**_ to a particular user set, *S*. Step 4 is used as a counter for *t* and Step 5 will repeat Step 1.

The cache_update function is used to update the caching contents. It consumes (as input) the list *L* of contents with extracted features and produces (as output) the prioritized caching content to be placed in F-AP. Step 1 selects the appropriate map size: horizontal *x* and vertical *y* dimensions of the map. Step 2 defines the color intensity of map nodes to depict classes for low, medium, and high priority contents. Step 3 is used to run the SOMs algorithm after initializing weights *W*_*ij*_. The mapping of *n*_DB_ and *n*_MB_ on *L* to get *C*_*P*_ in the Step 4. The map nodes with dark blue *n*_DB_ and medium blue *n*_MB_ color showing high priority and medium priority content as *L*. *C*_*P*_ are the prioritized contents to be placed in cache.

Serve_UE_Phase function is used to serve a user request  *r*_*k*_. It takes regional user set and assigned edge node as input and activate to serve for an incoming request. If there is a cache hit in Step 1, it will serve *r*_*k*_; otherwise, it will first update the cache and then serve *r*_*k*_.

## 4. Content Prioritization Results through Deep Learning

### 4.1. Self-Organizing Maps

Multimedia content needs to be prioritized with considerable views, likes, dislikes, and comments. SOMs algorithm learns from the given dataset and displays on the map by the grid of nodes. The degree of the relationship between data points is shown through the color intensity. As a proof of concept, a tool is implemented using Python programming language and is exploited through a Jupyter notebook in Google Colab. As mentioned earlier, the four input vectors, i.e., views, likes, dislikes, and comment_count, are selected utilizing the dataset to simulate. The feature scaling is achieved through the MinMax scaler. To train the SOMs algorithm, the tuning parameters with their values are simulated through the tool and shown in Table 1.

**Figure 7:**
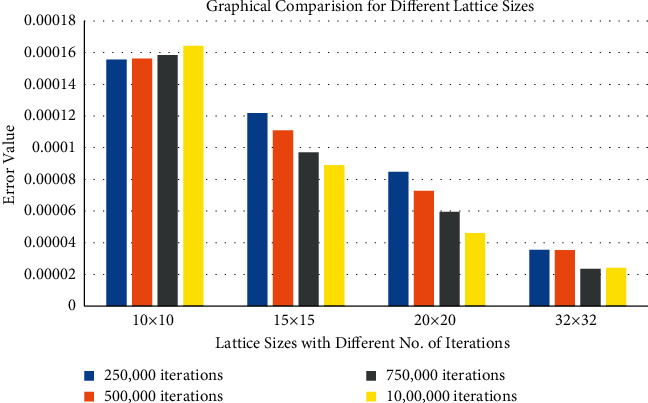
Comparison of different lattice sizes with the errors values by selecting different number of iterations.

To select an appropriate lattice size, different lattice sizes are tested by the hit and trial method to validate the formula. The experiment shows that the batch training yields many exact results; however, it is a bit slower than the random training. The recorded data are shown in Table 2. A histogram also represents the recorded data in Figure 7. On *x*-axis, different lattice sizes (i.e., 10 × 10, 15 × 15, 20 × 20, and 32 × 32) with different number of iterations (i.e., 250,000, 500,000, 750,000, and 1,000,000) are shown by different colors. On the *y*-axis, the error values are displayed (given in Table 2). Evaluating the data carefully proves that the error values are recorded minimum on the lattice size 32 × 32 with 750,000 iterations; therefore, this lattice size is considered appropriate.

**Figure 8:**
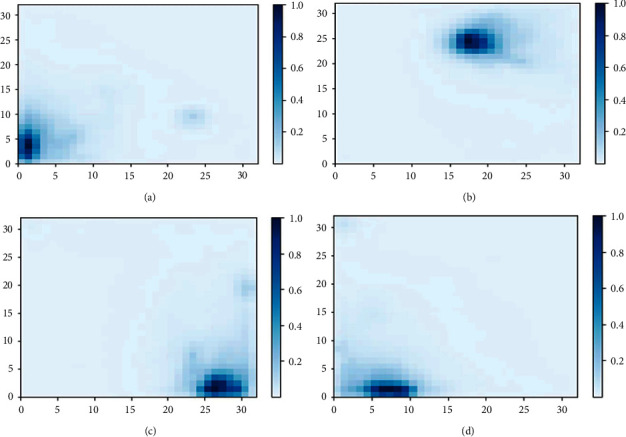
The color scales for different iterations at lattice size 32 × 32 are shown. (a) Output map at 250,000 iterations. (b) Output map at 500,000 iterations. (c) Output map at 750,000 iterations. (d) Output map at 1,000,000 iterations.

The map's outputs of different iterations for lattice size 32 × 32 are shown in Figure 8. The color scale for iterations 250,000, 500,000, 750,000, and 1,000,000 are shown in Figures 8(a)–8(d), respectively. Nodes with color (values) range from 0.8 to 1.0 (dark blue) which represent the group of data having high priority contents (maximum number of views, likes, dislikes, and comments). The medium priority contents are represented with light bluish color nodes ranging from 0.4 to 0.8. The medium priority contents follow the high priority contents. The remaining nodes with color values below 0.4 are considered the least priority contents and must not be deliberated in the caches.

**Figure 9:**
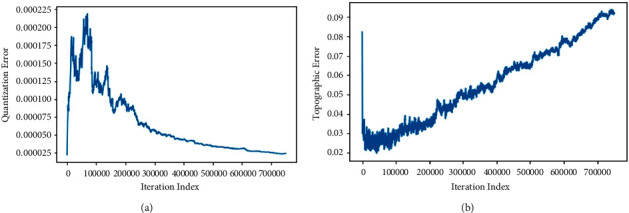
(a) Quantization error to check the validity of the data and (b) topographic error to determine how well the topology of the map is preserved at 750,000 iterations.

After the SOMs algorithm's training through different lattice sizes, the QE needs to be extracted to check the validity of the data. According to the data described in Table 2, the best map among all is 32 × 32 lattice-sized map with 750,000 iterations. The QE value is recorded even less than 0.000024 as shown by the graph in Figure 9(a). It is not reduced any further after 0.000024 QE value. The TE is plotted by Figure 9(b) to determine how well the topology of the map is preserved at 750,000 iterations. The TE value at this point is logged as 0.092. Although the TE is recorded a little bit higher but its value, together with QE value (i.e., 0.0000235) plotted the most appropriate map after 750,000 iterations. By taking into account the curves shown in Figures 9(a) and 9(b), it can be analyzed that the map is trained efficiently and delivers precise results.

After selecting the most appropriate lattice map (32 × 32) in 750,000 iterations, the data points located below the dark blue region are mapped onto the videos' data frame. Similarly, the light-blue color region is also mapped to get medium prioritized content.  Table 3 is used to describe the mapping data of high priority contents from one of the nodes from the dark blue region (27, 2). Also, it shows twelve highly trending videos (in rows from 0 to 11) with respect to views, likes, dislikes, and comment_count (in columns). These videos are considered as a high priority for trending according to the input parameters provided in the dataset.

The rest of the nodes from bluish-white to white are located in the lighter region and can be ignored because this region contains the least priority content.

### 4.2. Quantum Self-Organizing Maps

**Figure 10:**
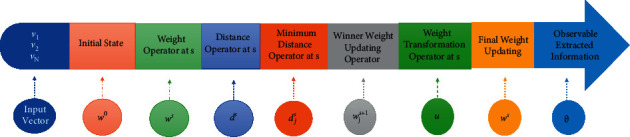
The general QuSOM structure contains gate array.

Quantum-SOM (QuSOM) has a different learning method than SOM. The number of presynaptic neurons corresponds to the number of neurons in both layers and interconnections between them when designing the QuSOM layout, which comprises the number of all model parameters and the set of potential data classifications. There is only one connection between a neuron in the input layer and a neuron in the output layer. QuSOM attracts all vectors of *v*, (*v*(*i*) ∈ *i*, *i* = 1, 2, ..., *N*), just once. The competitive and weight update is accomplished through a series of procedures, which is a parallel processing capability. As a result, in QuSOM, the traditional repetitive learning procedure is modified to learn only once.  Algorithm 2 of the QuSOM is as follows:

The QuSOM, in QC, may shift research directions in the artificial neural network (ANN) field depending on the computation environment and application property [38]. The parallelism aspect of the QuSOM is its most intriguing feature. A quantum mechanics computer can exist in a state of superposition and perform several operations simultaneously. A QuSOM gate array is depicted in Figure 10 as a schematic. The register's initial state is on the left, and time moves from left to right. The *W*^*s*^ gate is a weight operator at *s*; *D*^*s*^ gate is a distance operator at *s*; *d*^*s*^(*i*, *j*_min_) is a Grover searching oracle; *W*^*s*+1^ is a winner weight updating operator at *t*; U is a weight transformation operator at *s*, *u* = Q*W*^*t*+1^; *ϑ* is an observable extracted information from register, according to the above summarized QuSOM algorithm. The operations of these transformation and operation matrices are used to create QuSOM. The vectors are only entered into the map once, and the output (weight) should converge if the sequence is repeated.

In the traditional meaning of computation, putting all parameters in inputs as neurons may be unfeasible, and QuSOM operation will be time consuming due to parallelism. *N* = 2 input vectors with *M* = 4 total input items and *P* = 2 prototypes, for example, and the number of neurons in both input and output layers should be 2 × 4 × 2 = 16. This figure is four times that of SOM. Fortunately, this is not an issue in quantum computing. Quantum theory's peculiar properties can be used to express information with a neuron number of exponential capacity. The number of neurons is exponentially decreased to log2 for the input signals, *v*(*i*, *k*), *i* = 1, ..., *Z*, *k* = 1, ..., *Y*, *j* = 1, ..., *P*, by adopting quantum representation (MxNxP). QuSOM requires only 4 qubits in the example above. The input data from YouTube streaming may be more than 4 vectors with 40960 elements in some edge caching systems. The SOM configuration was used with 4 neurons in the input layer and 32 × 32 = 1024 neurons in the output layer. The network might be configured with 27 quantum input/output neurons, or qubits, representing roughly 41943040 SOM neurons using QuSOM. So, it can be determined that the gain difference in terms of computation and time consumption between the deep learning method based on quantum computing and conventional method as the measures of conventional computing are almost four times the quantum computing that clearly shows extensive use of resources in conventional computing.

### 4.3. Simulation Results for Quantum Phenomenon in Caching

**Figure 11:**
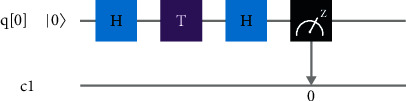
The circuit to produce rotation of the particles is shown from IBM QASM Simulator.

For the experiments, a cloud-based QC system (from IBM Quantum Experience) is used. The IBMQ_QASM_Simulator is basically a simulator backend, allows sampling circuits with a 32 qubits processor. The circuit to produce rotation of the particles is shown in Figure 11. It is a serially wired circuit comprising three gates (two Hadamard and one *T* gate). The respective circuit is used to act on qubit │0〉 and is known as a standardized measurement operator along the *z*-axis. The primary (first) wire, labeled as *q*[0], is a quantum wire representing the passage of time. It is not considered a physical wire. The gates are applied in unit time. The second wire, labeled as c1, is a classical wire, and the output from the quantum computer is determined once the measurement is applied. The vertical arrow from the measurement operator shows that the information is now retrieved from the quantum regime to the classical regime.

**Figure 12:**
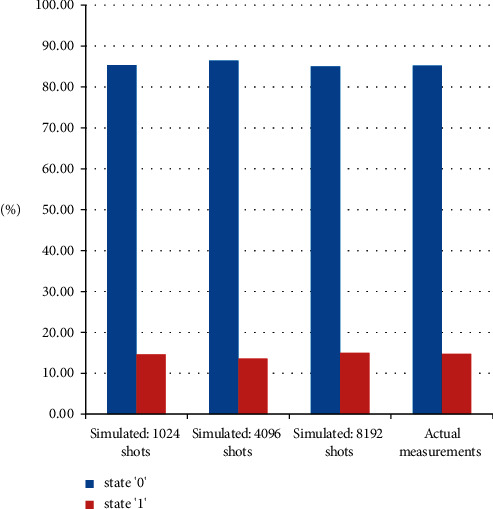
The simulated resultant probabilities with different number of shots, i.e., 1024 shots; 4096 shots; and 8192 shots; and the actual probability measurements are shown.

To evaluate circuit on the simulator, a parameter *number of shots* is needed to set before execution. The *number of shots* is simply a parameter having a value that determines the number of iterations, representing the number of times a quantum circuit executes. With the increase in the *number of shots*, the probability values of spin-up and spin-down are improved. The resultant probabilities are demonstrated by Figure 12 with different numbers of shots (i.e., 1024, 4096, and 8192 shots) and actual measurements. The horizontal axis of the histogram represents the computational basis states 0 and 1. The vertical axis represents the probability of observing that basis state. The histogram in Figure 12 also represents the exact probability measurement of the basis states.

As clearly depicted from Figure 12, there is a slight difference between the actual and simulated results. The first and last simulated results are realized by 1024 and 8192 shots, respectively. The probability measurement difference is reduced as compared with the actual result. The probability measurements are close to the actual result by 8192 shots. The results are concluded (by comparing actual and simulated) in Table 4.

All the prioritized contents are grouped in the form of color clusters. The color intensity of the nodes in the feature map makes it easier to prioritize the content. The nodes with less intense color illustrated the low prioritized content. The selection of highly prioritized and medium prioritized contents is achieved conveniently through the SOMs algorithm. The algorithm took less time and computational overhead than other DL algorithms. The use of TLSQP facilitates the overall management of most requested content within the edge for providing an instantaneous content delivery response. The concept of storing content in QMM by employing this quantum phenomenon is entirely unconventional and challenging at the same time. Nevertheless, its advantage overshadows other conventional approaches of the classical regime in terms of storage capacity and processing speed due to its unusual properties, i.e., quantum parallelism.

## 5. Discussion

The framework is basically a merger of a DL agent deployed at the network edge and a QMM. Firstly, the DL agent prioritizes caching contents via SOMs algorithm, and secondly, the prioritized contents are stored in QMM using TLSQP. The study of QuSOM follows the development tendency of ANN. The adaptation of ANN in the parallel computing environment will be interesting for both ANN and QC, especially for the simulation of human learning and memorizing features by using more powerful computing tools. In Kohonen's SOM, the learning and weight updating are organized in the same sequence. This sequence is like the human's repeated learning manner. In QuSOM, due to its once learning property, the weight updating is managed separately with learning and updating. This manner may appear more similar to human's once learning way. QuSOM has the same convergence property as Kohonen's SOM, but its time and space complexities are more simplified. To verify the valuation and efficiency of the algorithm, in this study, we have compared the gain difference in terms of computation and time consumption between the deep learning method based on quantum computing and conventional method which can be summarized as the measures of conventional computing are almost four times the quantum computing.

To verify the algorithm, we conduct extensive experiments to demonstrate that the algorithm improves the generalizability of the conventional SOM through optimization and is robust to the choice of hyperparameters, as listed in Table 5.

A hybrid approach was used for this work: one (i.e., SOM) for classifying the videos dataset and a second (i.e., TLSQP) for the storage of prioritized content. It can be inferred that the proposed framework DL-QC is deployed for edge caching in sensor network traffic to improve the prioritization and storage processes by exploiting the capabilities of DL and QC. Dataset has been selected that incorporates multimedia content that has been infrequently used in the past for other studies. The dataset contains four features and 40960 samples. Google Colab and IBM's Quantum Experience are utilized in this work with high certainty because of their capabilities of creating legitimate outcomes that mirror certain domains of intelligence and quantum. The gathered information has been recorded for prioritization levels and will notify the prioritized cases to the QMM storage through TLSQP, whereas the most minor prioritized cases will be removed with a higher accuracy rate. The DL algorithm SOM is precisely applied to the identified dataset for prioritization in a 32 × 32 lattice size. The selected DL classifiers accomplished the particular task with accuracy and precision, as discussed above. It will help characterize the high priority and medium priority network traffic to ensure the optimized caching services in the edge computing environment, and TLSQP ensures maximum data storage in QMM. Due to the research scope, some common types of multimedia content parameters have been selected, but in the future, more categories of innovative content and features in the DL-QC environment can be incorporated to better understand and cope with the identified issues. Furthermore, innovative algorithms can also be designed, or existing ones can be modified to prioritize and storage than already discussed to improve efficiency and accuracy. Moreover, the QuSOM can be replicated on conventional computers as well as quantum computers provided that the availability of resources to understand the results better.

## 6. Conclusion

The caching content's storage is mainly the primary source of immediate delivery responses. This research work has presented an intelligent DLAQC framework for updating the EC content in F-RANs. The caching content is logically prioritized through an intelligent DL agent in the network edge using the SOMs algorithm. The caching content is physically stored in QMM, exploiting the TLSQP phenomenon to update the caches and provide ample content storage for immediate delivery response against the unpredicted amount of static and dynamic user requests. The framework is evaluated using multimedia content and provides effective outcomes, especially by reducing computation overhead and time. The purpose is to form clusters to separate high, medium, and low-prioritized contents in an unsupervised manner. SOMs algorithm is staunchly suitable to pick up contents in colored cluster form without reducing the dimensionality of the feature space.

While the experiments have been conducted for multimedia content only, other contents can be considered, especially in IoT-based scenarios where unpredictable amounts of static and dynamic requests are generated day by day. EC is capable of handling each request immediately; it is still challenging and can be considered to explore further. Besides, innovative algorithms can also be designed, or existing can be modified to prioritize and storage than already discussed to get better efficiency and accuracy. Moreover, the QuSOM can be replicated on conventional computers as well as quantum computers provided the availability of resources to better understand the results.

## Figures and Tables

**Algorithm 1:**
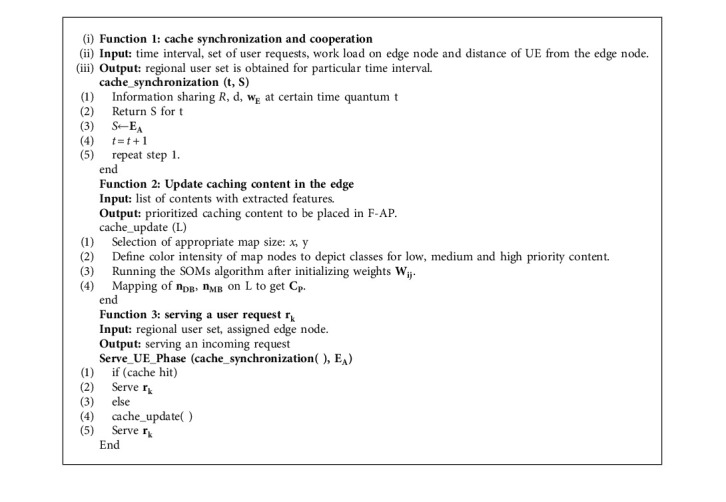
Cache content management.

**Algorithm 2:**
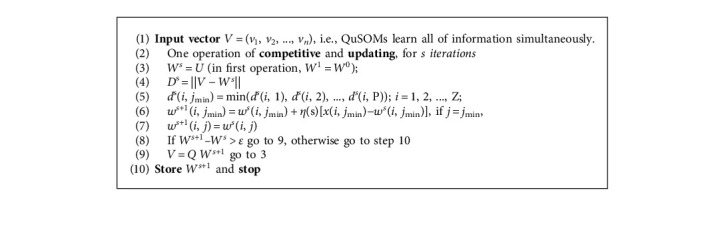
Learning through quantum self-organizing maps.

**Table 1:** Tuning parameters and their values.

Tuning parameters with symbols	Values
*x*-dimension of the lattice (*x*)	32
*y*-dimension of the lattice (*y*)	32
Learning rate (**L**_**t**_)	0.1
Initial spread value (*σ*_0_)	1.0

**Table 2:** The errors' values with respect to different lattice sizes and the number of iterations.

Dimensions of lattice	No. of iterations			
	250,000	500,000	750,000	1,000,000
10 × 10	0.0001555	0.0001563	0.0001584	0.0001643
15 × 15	0.0001218	0.0001110	0.0000970	0.0000890
20 × 20	0.0000847	0.0000727	0.0000594	0.0000460
32 × 32	0.0000355	0.0000353	0.0000235	0.0000241

**Table 3:** The data of the Mapping Dark Blue Node (27, 2).

	Views	Likes	Dislikes	Comment_count
0	167997997.0	4281819.0	276626.0	453206.0
1	173478072.0	4360121.0	283961.0	460299.0
2	179045286.0	4437175.0	291098.0	466470.0
3	184446490.0	4512326.0	298157.0	473039.0
4	190950401.0	4594931.0	305435.0	479917.0
5	196222618.0	4656929.0	311042.0	485797.0
6	200820941.0	4714942.0	316129.0	491005.0
7	205643016.0	4776680.0	321493.0	496211.0
8	210338856.0	4836448.0	326902.0	501722.0
9	217750076.0	4934188.0	335462.0	509799.0
10	220490543.0	4962403.0	338105.0	512337.0
11	225211923.0	5023450.0	343541.0	517232.0

**Table 4:** Comparison of actual and simulated probability measurements.

Basic states	Probability measurements			
	Actual (%)	Simulated (1024 shots)	Simulated (4096 shots) (%)	S (%) imulated (8192 shots)
0	85.35534	86.426	85.01	85.242
1	14.64466	13.574	14.99	14.758

**Table 5:** Comparison with previous research.

Research	Deployed algorithm	Performance measurements	Results		
[39]	Multiagent deep reinforcement learning (MADRL)	Caching reward	21%		
		Cache hit rate	Highest		
		Traffic load	43%		
	Multiagent actor-critic (MAAC)	Caching reward	56%		
		Cache hit rate	Higher		
		Traffic load	45%		
	Deep reinforcement learning (DRL)	Caching reward	43%		
		Cache hit rate	Lower		
		Traffic load	36%		
	Least recently used (LRU)	Caching reward	34%		
		Cache hit rate	Lowest		
		Traffic load	12%		

[40]	Personalized edge caching system (PECS)	Deep packet inspection	Top-down analysis (network level) and bottom-up analysis (user level)		

[41]	One-dimensional convolutional neural network (ODCNN) Self-Organizing Map (SOM)	Accuracy rate	99.8%		

[42]	Support vector machine	Accuracy	0.984		
		Precision	0.984		
		Recall	0.983		
		F1 score	0.981		
	Logistic regression	Accuracy	0.983		
		Precision	0.982		
		Recall	0.983		
		F1 score	0.983		
	K-nearest neighbors	Accuracy	0.984		
		Precision	0.983		
		Recall	0.984		
		F1 score	0.984		
	Isolation forest	Accuracy	0.870		
		Precision	0.969		
		Recall	0.973		
		F1 score	0.919		

Proposed	Self oranizing map (SOM)	Quantization error	0.000024		
		Topographic error	0.092		
		QE + TE	0.0000235		
	Two-level spin quantum phenomenon (TLSQP)	Basic states	0	OV = 85.4%	PV = 85.2%
			1	OV = 14.6%	PV = 14.8%

## Data Availability

A publicly available dataset is used for this study (Trending YouTube Video Statistics).

## Supplementary Materials

Supplementary Materials The data used to support the findings of this study are provided in “Annexure.”
