# Supplementary material for: Edge Caching in Fog-Based Sensor Networks through Deep Learning-Associated Quantum Computing Framework
Source: Comput Intell Neurosci. 2022 Jan 7;2022:6138434. doi: 10.1155/2022/6138434 (PMC8759837; doi:10.1155/2022/6138434)
Supplement: Supplementary Materials — The data used to support the findings of this study are provided in “Annexure.” [file 6138434.f1.docx]

Annexure

| **Full Form** |  | **Acronyms** |  |
| --- | --- | --- | --- |
| Fog Computing |  | FC | 1. Acronyms used in Proposed Study |
| Mobile Edge Computing |  | MEC |  |
| Internet of Things |  | IoT |  |
| Quality of Service |  | QoS |  |
| Quantum Computing |  | QC |  |
| Edge Caching |  | EC |  |
| Deep Learning |  | DL |  |
| Quantum Memory Module |  | QMM |  |
| Artificial Neural Network |  | ANN |  |
| Self Organizing Maps |  | SOMs |  |
| Quantum Self Organizing Map |  | QuSOM |  |
| Two-Level Spin Quantum Phenomenon |  | TLSQP |  |
| Base Band Unit |  | BBU |  |
| Fog Computing based Radio Access Networks |  | F-RANs |  |
| Remote Radio Heads |  | RRHs |  |
| Cloud Radio Access Networks |  | CRANs |  |
| Heterogeneous Cloud Radio Access Networks |  | HCRANs |  |
| Fog Access Points |  | FAPs |  |
| Base Stations |  | BS |  |
| Deep Learning Associated Quantum Computing |  | DLAQC |  |
| Stern Gerlach Experiment |  | SGE |  |
| Quantum Paralellism |  | QP |  |
| Quantization Error |  | QE |  |
| Topographic Error |  | TE |  |
| Mixed-Integer Nonlinear Programming |  | MINL | 1. Acronyms used in the Included Literature |
| Deep Reinforcement Learning |  | DRL |  |
| Federated Learning |  | FL |  |
| Deep Neural Network |  | DNN |  |
| Failure Modes and Effects Analysis |  | FEMA |  |
| Multiple Travelling Salesman Problem |  | MTSP |  |
| Adaptive Neuro-Fuzzy Inference System |  | ANFIS |  |
